# Mycolactone enhances the Ca^2+^ leak from endoplasmic reticulum by trapping Sec61 translocons in a Ca^2+^ permeable state

**DOI:** 10.1042/BCJ20210345

**Published:** 2021-11-23

**Authors:** Pratiti Bhadra, Scott Dos Santos, Igor Gamayun, Tillman Pick, Clarissa Neumann, Joy Ogbechi, Belinda S. Hall, Richard Zimmermann, Volkhard Helms, Rachel E. Simmonds, Adolfo Cavalié

**Affiliations:** 1 Center for Bioinformatics, Saarland University, Saarland Informatics Campus, 66123 Saarbrücken, Germany; 2 Department of Microbial Sciences, School of Biosciences and Medicine, University of Surrey, Guildford, Surrey GU2 7XH, U.K.; 3 Experimental and Clinical Pharmacology and Toxicology, Saarland University, 66421 Homburg, Germany; 4 Medical Biochemistry and Molecular Biology, Saarland University, 66421 Homburg, Germany

**Keywords:** calcium imaging, calcium signalling, molecular docking, mycolactone, Sec61, translocon

## Abstract

The *Mycobacterium ulcerans* exotoxin, mycolactone, is an inhibitor of co-translational translocation via the Sec61 complex. Mycolactone has previously been shown to bind to, and alter the structure of the major translocon subunit Sec61α, and change its interaction with ribosome nascent chain complexes. In addition to its function in protein translocation into the ER, Sec61 also plays a key role in cellular Ca^2+^ homeostasis, acting as a leak channel between the endoplasmic reticulum (ER) and cytosol. Here, we have analysed the effect of mycolactone on cytosolic and ER Ca^2+^ levels using compartment-specific sensors. We also used molecular docking analysis to explore potential interaction sites for mycolactone on translocons in various states. These results show that mycolactone enhances the leak of Ca^2+^ ions via the Sec61 translocon, resulting in a slow but substantial depletion of ER Ca^2+^. This leak was dependent on mycolactone binding to Sec61α because resistance mutations in this protein completely ablated the increase. Molecular docking supports the existence of a mycolactone-binding transient *inhibited* state preceding translocation and suggests mycolactone may also bind Sec61α in its *idle* state. We propose that delayed ribosomal release after translation termination and/or translocon ‘breathing' during rapid transitions between the *idle* and *intermediate-inhibited* states allow for transient Ca^2+^ leak, and mycolactone's stabilisation of the latter underpins the phenotype observed.

## Introduction

Mycolactone is the exotoxin produced by *Mycobacterium ulcerans* (*M. ulcerans*) that is central in the aetiology of Buruli ulcer (BU), a chronic necrotising skin infection [1–3]. Most pathogenic strains isolated from BU patients living in West Africa, where the disease is most prevalent, produce mycolactone A/B. Other congeners also exist, produced by pathogenic strains found in Australia (mycolactone C) and so-called ‘ancestral' strains. Mycolactone A/B is the most toxic *in vitro* and is a polyketide composed of an invariant 12-membered lactone ring to which two polyketide-derived, unsaturated acyl side chains are attached [1,3]. The shorter ‘Northern' chain is invariant, while the longer ‘Southern' Chain gives rise to the various mycolactone congeners. Like all mycolactones, mycolactone A/B is a 3 : 2 rapidly equilibrating mixture of isomers around the second double bond in the Southern fatty acid tails [4]. Structure-activity studies have proposed that mycolactone B is the active isoform [5].

Infection with *M. ulcerans* initially produces a painless swelling or nodule. The immunosuppressive properties of mycolactone enable the disease to progress without inflammation and fever, leading eventually to development of the necrotic lesions in cutaneous and subcutaneous tissues that are characteristic of BU [6]. Many reports support mycolactone as driving the pathogenic sequelae of BU that comprise skin ulceration, coagulative necrosis, local hypoesthesia and suppression of immune responses. Furthermore, molecular targets for mycolactone have been identified and proposed to be associated with its hallmarks [2,3]. Binding and activation of type 2 angiotensin receptors II initiates a signalling cascade that culminates in the activation of TWIK-related arachidonic acid-stimulated K^+^ channel in neurons [7]. The resulting hyperpolarisation of neurons was proposed to mediate the analgesic effects of mycolactone. In cell free assays, mycolactone binds to the Wiskott–Aldrich syndrome protein (WASP) and neural WASP (N-WASP), which are scaffold proteins involved in the remodelling of the actin cytoskeleton [8,9]. By interacting with WASP/N-WASP, mycolactone likely initiates an uncontrolled assembly of actin and defective cell–matrix adhesion. Notably, the down-regulation of multiple mediators of inflammation correlates solely with the continuous presence of mycolactone even after completion of antibiotic therapy against *M. ulcerans*, providing a link between mycolactone and immunosuppressive effects [10].

Subsequently, investigations using cell-free protein synthesis showed that mycolactone inhibits the Sec61-dependent protein translocation into the endoplasmic reticulum (ER) [11]. Furthermore, the blockade of Sec61-dependent translocation by mycolactone has been linked to inhibition of T cell activation and antigen presentation, providing a molecular basis for the mycolactone-induced immunosuppression [12,13]. The effects of mycolactone on ER function are however much broader, due to the Sec61-dependent nature of many ER-resident proteins. However, while it has been shown that mycolactone down-regulates steady state mRNA levels of luminal Hsp70-type chaperone binding immunoglobulin protein (BiP; HSPA5) in dendritic cells [14], proteomic data indicates that BiP expression may be sustained [13,14, Hall, Hsieh et al., unpublished). Furthermore, inhibition of Sec61-mediated translocation by mycolactone induces an integrated stress response via translational activation of activating transcription factor 4 [15]. Thus, a consequence of the blockade of Sec61 translocons is a stress response that eventually induces apoptosis and underlies cytotoxic effects of mycolactone. A forward genetic screen in HCT116 cells for mutations that confer resistance to mycolactone-induced toxicity resulted in >30 clones, all of which contained at least one heterozygous mutation in the gene encoding Sec61α, SEC61A1 [15,16], indicating that Sec61α is the prime target of mycolactone. Mutations were clustered at a number of sites in Sec61α (D60, R66, S71, G80, S72 and Q127) [15,16]. Several of these mutations have been studied in detail and have been shown to reverse the binding to and/or biological effects of mycolactone [12,15–17].

Sec61α plays a central role in the translocation of nascent polypeptides emerging from the translating ribosome. To accomplish translocation, the Sec61 complex undergoes substantial conformational changes. McKenna et al. (2016) provided the first biochemical evidence that the binding of mycolactone to Sec61α also induces conformational changes of the pore geometry [18]. Several crystal and cryo-electron microscopy structures have revealed the conformation of the native ribosome–Sec61 complex in the non-translocating (*idle*) state [19–21]. Here, the lateral gate of the Sec61α is closed and the so-called ‘plug’ helix of Sec61α seals the channel toward the lumenal side [22]. During translocation, the conformation of ribosome-Sec61 complexes is significantly different. Here, the plug is displaced to enable translocation and the lateral gate remains open for membrane access of the signal peptide or transmembrane domains of the nascent precursor polypeptide [23]. Recently, Gérard et al. [16] determined the structure of mammalian ribosome-Sec61 complexes inhibited by mycolactone via electron cryo-microscopy, where the Sec61α channel is trapped in a putative *intermediate* state. In this state, Sec61α is in a conformation where the cytosolic side of the lateral gate is open, whereas the plug still occupies the channel pore in the lumenal side. Notably, this conformation closely resembles that of yeast Sec61 translocons bound to Sec62 and Sec63 [24,25], suggesting that this pose is adopted during native translocation at least in lower eukaryotes. The structure provides novel insight into the molecular mechanism of mycolactone as an inhibitor. Notably, mycolactone binds with weaker affinity to translocons from cells carrying resistance mutations in Sec61α [16]. Here, molecular dynamics simulations suggested that the single amino acid mutations modulate the conformational dynamics of Sec61α and thereby disfavour the mycolactone-bound conformation.

It is striking that the molecular targets of mycolactone have been associated with key functions in the Ca^2+^ homeostasis. For instance, Sec61α translocons behave as Ca^2+^ permeable channels that support a Ca^2+^ leak from ER that is regulated by BiP [26]. Mycolactone may also interact with WASP/N-WASP leading to actin assembly [9], potentially impacting Ca^2+^ homeostasis because actin polymerisation in the cell periphery prevents the coupling between the ER and store-operated Ca^2+^ channels, reducing the Ca^2+^ entry into the cell [27]. On the other hand, actin polymerisation enhances the Ca^2+^ mobilisation from ER that is induced by the sarco/endoplasmic reticulum Ca^2+^-ATPase (SERCA)-inhibitor thapsigargin (TG) [28]. Finally, as a lipophilic substance, mycolactone interacts with phospholipids and hence may perturb cholesterol-rich membranes more generally [29–31].

Thus, while it can be hypothesised that mycolactone might distort Ca^2+^ homeostasis in the cell, until now this area has been little explored. An early study reported a dose-dependent increase in cytosolic Ca^2+^ levels in L929 cells exposed to mycolactone [32]. Later, it was shown that mycolactone-mediated hyperactivation of Lck in Jurkat cells resulted in depletion of intracellular Ca^2+^ stores and T cell receptor down-regulation [33]. As in the study with L929 cells, increased cytosolic Ca^2+^ levels were also found in resting T cells exposed to mycolactone. Additionally, mycolactone reduced the store-operated Ca^2+^ entry and the Ca^2+^ mobilisation induced by the non-selective iononophore ionomycin (IONO) in these T cells [33]. In the present study, we focused on the effects of mycolactone on Ca^2+^ leak from the ER. Using mycolactone-resistant mutants of Sec61α [15–17], we show in Ca^2+^ imaging experiments that mycolactone specifically enhances the Sec61-mediated Ca^2+^ leak from ER. By docking analysis, we further explore potential interaction sites for mycolactone with Sec61 translocons at the molecular level. These analyses support the mechanistic model in which mycolactone stabilises the *intermediate* (mycolactone-bound/mycolactone-inhibited) conformation of the ribosome-translocon complex, which in turn generates a Ca^2+^ leak from the ER.

## Materials and methods

### Cell lines

The experiments were done with a HEK-293 cell line that stably expresses the FRET-based D1ER sensor in the ER lumen (D1ER-HEK) as well as with HCT116 cells that express Sec61α mutants (D60G, R66K, S71F, S82Y, Q127K) and RAW 264.7 cells [11,15,16,34]. D1ER was kindly provided by R. Y. Tsien [35] and stably expressed in HEK-293 cells to obtain the D1ER-HEK cell line [34]. D1ER-HEK cells were cultured under selection with G418 (Minimal Essential Medium, MEM (Gibco), 10% FBS, 0.5 mg/ml G418). Wild-type (WT) HCT116 cells and those expressing Sec61 mutants were maintained in McCoy's 5A Medium (Gibco) and 10% FBS. HCT116 cells expressing Sec61 mutants (D60G, R66K, S71F, S82Y, Q127K) were generated by random mutagenesis with ENS in the DNA repair defective HCT116 cell line [15]. Prior to Ca^2+^ imaging experiments, HCT116 cells were plated on poly-l-lysine coated cover slips and transfected with ER calcium sensor ER-GCaMP6-150 using FuGENE HD (Promega Corp.). Experiments were carried out 1–2 days after transfection. ER-GCaMP6-150 was kindly provided by J. de Juan-Sanz and T. A. Ryan [36]. The murine macrophage cell line RAW 264.7 was routinely cultured in high glucose Dulbecco's Modified Eagle's Medium (DMEM) supplemented with 10% FBS [11]. For Ca^2+^ imaging experiments, D1ER-HEK and RAW 264.7 cells were transferred to poly-l-lysine coated cover slips and maintained in culture for 2–3 days. Cell culture was carried at 37°C in a humidified environment with 5% CO_2_.

### Reagents and cell treatment

Synthetic mycolactone (MYC) A/B was kindly provided by Y. Kishi [37]. Aliquots containing 0.5 mg/ml mycolactone in DMSO were maintained at −80°C in the dark. Before the experiments, the mycolactone stock solution was diluted directly in complete culture medium or recording solution to the desired concentrations. Thapsigargin (TG, Thermo Fisher Scientific) and ionomycin (IONO, Thermo Fisher Scientific) were dissolved in DMSO to obtain 1 mM and 10 mM stocks, respectively. TG and IONO stock were maintained at −20°C in the dark and dilutions to the desired concentrations were made directly in the recording solution just before experiments.

FURA-2 AM (Thermo Fisher Scientific) was dissolved in DMSO to obtain a 1 mM stock solution, which was subsequently dissolved to 4 µM in a Ca^2+^ containing solution (140 mM NaCl, 5 mM KCl, 1 mM MgCl_2_, 1 mM CaCl_2_, 10 mM glucose, 25 mM HEPES, pH 7.2). Cells were loaded with FURA-2 prior imaging experiments by incubation with the solution containing 4 µM FURA-2 AM for 20 min.

MYC, TG and IONO were routinely applied as 2× solutions to the bath at a ratio of 1 : 1 to avoid problems arising from slow mixing. MYC was added to the culture medium at the desired concentrations and cells were cultured for 1, 6 and 18 h before imaging recordings. for the so-called ‘online' treatment in Ca^2+^ imaging experiments, MYC was applied to the bath while the recording of images was running (Figure 3A). When cells were transfected with ER-GCaMP6-150, MYC was present in the culture medium containing the transfection reagents during the last 6 h before imaging recordings. Controls for all experiments were DMSO diluted to a similar extent as the highest concentration found in mycolactone treatments, i.e. 0.02–0.05% v/v DMSO in bath solution or culture medium.

### Live cell Ca^2+^ imaging

Ca^2+^ imaging experiments were carried out in the absence of Ca^2+^ in the bath solution to prevent Ca^2+^ entry from the extracellular space. Thus, the bath solution contained 140 mM NaCl, 5 mM KCl, 1 mM MgCl_2_, 0.5 mM EGTA, 10 mM glucose and 10 mM HEPES-KOH (pH 7.35). Using an iMIC microscope equipped with a polychromator V and the Live Acquisition Software (Till Photonics), we imaged cytosolic Ca^2+^ concentrations ([Ca^2+^]_cyt_) using FURA-2 (i) and Ca^2+^ concentrations in ER ([Ca^2+^]_ER_) with the Ca^2+^ sensors D1ER (ii) and ER-GCaMP6-150 (iii).

i)  **Imaging cytosolic Ca^2+^ with FURA-2.** FURA-2 was excited at 340 and 380 nm alternately. The emitted fluorescence was captured at 510 nm to obtain FURA-2 images at 340 and 380 nm excitation. FURA-2 image pairs containing 40–50 cells/frame were obtained every 3 s at a magnification of 40×. FURA-2 signals were measured in image pairs as F340/F380, where F340 and F380 correspond to the background-subtracted fluorescence intensity at 340 and 380 nm excitation wavelengths, respectively. [Ca^2+^]_cyt_ was calculated with the standard ratiometric equation [Ca^2+^]_free_ = βK_d_,((R − R_min_)/(R_max_ − R)), in which R = F340/F380 [38]. βKd, maximal and minimal F340/F380 (R_min_ and R_max_) were measured as previously described [34]. Results of FURA-2 measurements are presented either as F340/F380 ratios or as [Ca^2+^]_cyt_.
ii)   **Imaging ER Ca^2+^ with D1ER.** D1ER-HEK cells were exposed to 433 nm and the emitted fluorescence was split at 469 nm and 536 nm to obtain the CFP and citrine components, respectively [35]. The cell fluorescence was additionally passed through a dichrotome and projected on the chip of the microscope camera to obtain simultaneous CFP and citrine images. The FRET ratios were calculated from background-subtracted CFP and citrine image pairs as F_Citrine_/F_CFP_, where F_Citrine_ and F_CFP_ represent the citrine and CFP fluorescence intensities at 536 nm and 469 nm, respectively. To allow the recording of [Ca^2+^]_cyt_ and [Ca^2+^]_ER_ in the same cells, the FURA-2 and D1ER filter sets were exchanged automatically [34]. In each cycle, FURA-2 images were first recorded at 340 and 380 nm excitation and D1ER images at 469 and 536 nm emission afterwards. D1ER and FURA-2 images containing 10–15 cells/frame were obtained at 60× magnification every 10 s. Results of D1ER and FURA-2 measurements that reflect the dynamic of [Ca^2+^]_ER_ and [Ca^2+^]_cyt_ in the same cells are presented as F_Citrine_/F_CFP_ and F340/F380 ratios, respectively.
iii) **Imaging ER Ca^2+^ with ER-GCaMP6-150.** Cells transfected with ER-GCaMP6-150 were exposed to 480 nm and the emitted fluorescence was collected at 515 nm [36]. Images with 3–10 cells/frame were recorded at a 60× magnification every 2 s. The ER-GCaMP6-150 fluorescence (F) was measured in background-subtracted images and normalised with respect to the fluorescence measured 2 min after starting recordings (F_0_). Results of measurements with ER-GCaMP6-150 that reflect the dynamic of [Ca^2+^]_ER_ are presented as F/F_0_ ratios.

### Homology modelling and molecular docking

The 476 amino acids long protein sequence of human Sec61α isoform 1 was retrieved from Uniprot (ID: P61619). Using homology modelling, structural models of human Sec61α were constructed using the following electron microscopy (EM) structures of Sec61α from *Canis lupus* (Uniport ID: P38377) as a template (99.8% sequence identity using global sequence alignment): *idle* conformation (PDB code 3J7Q with a resolution of 3.4 Å) [21]; *open* conformation (3JC2 with resolution of 3.6 Å) [23]; and the *inhibited* state of Sec61α in the presence of MYC (6Z3T with a resolution 2.6 Å) [16]. The structural information of the missing part of 6Z3T was obtained by homology modelling based on 2WWB (EM structure with resolution 6.48 Å). Homology modelling was performed using MODELLER 9.21 [39]. After sequence alignment of target and template, MODELLER was run locally with the automodel class to generate 50 different models. As structure 3JC2 is lacking structural information for the plug region, the plug region of the open conformation was modelled as loop. The homology model with the lowest discrete optimisation of potential energy (DOPE) score was selected as the final model and subjected to 1000 steps of energy minimisation, using the GROMACS package (version 5.0.7) [40] to relax side-chain atoms. For this, the protonation states of titratable amino acids were determined by the web server PDB2PQR [41] using the PROPKA method [42]. The total charge of Sec61α was +4e.

Two different isomers of MYC were used for docking, MYC B (E-isomer) and MYC A (Z-isomer) (see Supplementary Figure S1). Docking of MYC was conducted using AutoDock4.2 [43] to predict energetically favourable binding poses of the ligand inside or on the surface of WT human Sec61α in the idle, open and MYC-inhibited conformations. With respect to protein–ligand docking, MYC is a large compound containing 23 rotatable bonds. For each ligand, MYC A and B, the docking calculations were performed in two consecutive steps: in the first docking step, we adopted a relatively large grid box (100 Å × 100 Å × 126 Å) (see Supplementary Figure S2) covering the entire cavity of Sec61α in order to achieve an unbiased approach to determine potential binding sites. The Lamarckian genetic algorithm was employed to search for favourable binding poses, with a population size of 150, 27 × 10^3^ generations and 25 × 10^5^ energy evaluations. All other docking parameters for protein and ligand were set to the default values of AutoDock. 2000 individual docking results were clustered according to a threshold for structural similarity of 2.0 Å RMSD. In each cluster, the representative conformation was set to the one with the lowest binding free energy for that cluster. Three independent sets of 2000 docking runs each were conducted in the first stage.

In the second docking stage, the size of the grid box was reduced to 90 Å × 80 Å × 80 Å dimension (idle conformation), 80 Å × 90 Å × 80 Å dimension (intermediate conformation), and 80 Å × 80 Å × 90 Å (open conformation), respectively. This was done based on the populations of the most stable binding positions of the ligand. In the fine docking runs, more stringent parameters were used, namely 0.5 × 10^6^ generations and 100 × 10^6^ energy evaluations per run. In this stage, we executed five independent fine docking runs for the idle and intermediate conformations of Sec61α. The first step of docking to the open conformation of Sec61α showed a larger conformational diversity of MYC within the Sec61α channel pore than for the idle conformation, ten independent fine docking runs were performed for the open conformation. In each docking run we generated 50 docked conformations.

### Membrane-associated polysome profiling

RAW 264.7 cells grown to 80% confluency on 15 cm dishes were incubated with 31.25 ng/ml MYC or solvent control for 4 h at 37°C. Membrane-associated polysomes were prepared following the methods described in Potter and Nicchitta, 2002 [44], with modifications. Cyclohexamide (CHX) (100 μg/ml) was added 5 min prior to harvesting. Cells were washed once with ice-cold PBS containing 100 μg/ml CHX then harvested in KHM buffer (25 mM K-Hepes, pH 7.2, 400 mM KOAc, 25 mM Mg(OAc)_2_, 100 μg/ml CHX). Cells were pelleted by centrifugation at 280×***g*** for 3 min at 4°C, then resuspended in ice-cold KHM containing 0.03% digitonin and incubated on ice for 5 min. After centrifugation again at 280×***g*** for 3 min at 4°C, pellets were solubilised by incubation with KHM containing 2% digitonin, 1 mM PMSF and 40 U/ml RNase inhibitor. Lysates were centrifuged at 7500***g*** for 10 min at 4°C and layered onto a 10–60% sucrose gradient in KMH containing 0.1% digitonin and 100 μg/ml CHX. Polysome profiling was carried out as described previously [11]. Proteins in column fractions were acetone precipitated and separated by SDS PAGE on 4–15% gradient gels (Bio-Rad) and blotted onto Immobilon PVDF membranes (Merck). Blots were probed with mouse monoclonal anti-Sec61α (Santa Cruz Biotechnology) and rabbit polyclonal anti-Sec61β antibodies [45] and detected with ECL anti-mouse IgG and anti-rabbit IgG, HRP linked antibodies (GE Healthcare), respectively.

### Viability assay

Parental HCT116 cells were seeded onto 96 well plates at a density of 2 × 10^4^/ml and incubated overnight. Mycolactone (125 ng/ml) was added, and cells were incubated a further 18 h. To assess cell death, propidium iodide (Merck) was added to a final concentration of 0.3 µg/ml with Hoechst 33342 (ThermoFisher) at 4 µM. Cells were incubated for 30 min then imaged on a Nikon A1 confocal microscope. The percentage of propidium iodide-stained nuclei was quantified for at least three fields.

### Statistical analysis

**Figure 1.**
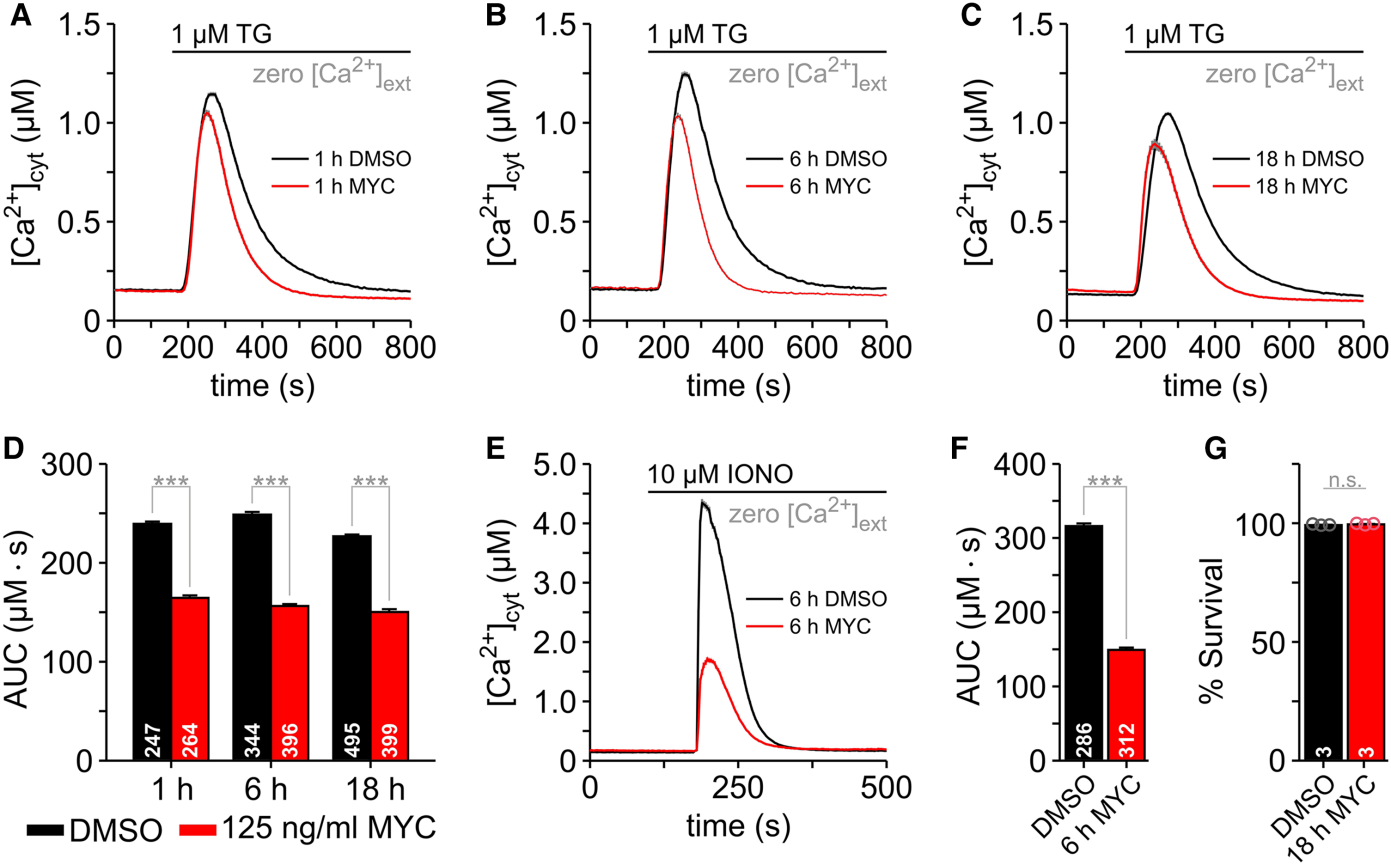
Mycolactone attenuates the Ca^2+^ mobilisation in HCT116 cells. Changes in cytosolic Ca^2+^ ([Ca^2+^]_cyt_) were imaged with FURA-2. HCT116 cells were treated with 0.05% DMSO or 125 ng/ml mycolactone (MYC) for 1 h (**A**), 6 h (**B**) and 18 h (**C**) before FURA-2 loading and Ca^2+^ imaging. To avoid Ca^2+^ entry into the cells, experiments were carried out in the absence of external Ca^2+^ (zero [Ca^2+^]_ext_). Thapsigargin (1 µM TG) was added to the bath solution to unmask Ca^2+^ leak from ER (**A**–**C**). The area under the curve (AUC) of the TG-induced Ca^2+^ transients was used as a measure of the mobilisable Ca^2+^ from intracellular TG-sensitive stores (**D**). The total amount of intracellular mobilisable Ca^2+^ was estimated by treating cells with ionomycin (10 µM IONO) (**E**). The AUC of the IONO-induced Ca^2+^ transients reflect the total amount of Ca^2+^ that is stored within the cells (**F**). The number of cells imaged in the experiments is given within the graph bars in **D** and **F**. Mean cell survival of HCT116 cells incubated for 18 h with 125 ng/ml mycolactone, stained with propidium iodide and Hoechst 33342 and imaged by confocal microscopy (**G**). Values represent the mean of three independent experiments. Data is presented as means ± SEM; n.s., non-significant; *** *P* < 0.001.

**Figure 2.**
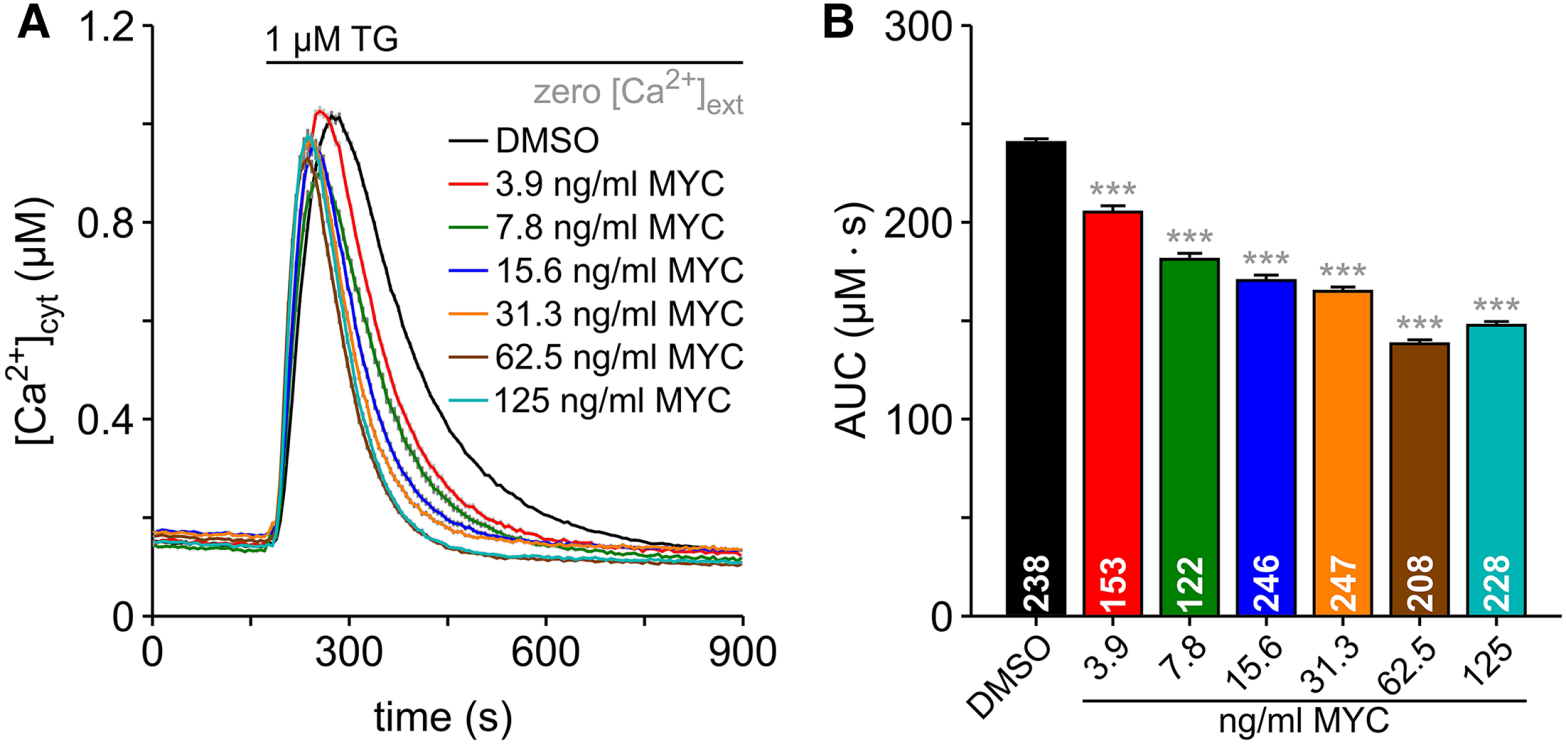
Dose-dependent effects of mycolactone on Ca^2+^ mobilisation in HCT116 cells. The dose-dependence of mycolactone (MYC) on the thapsigarin (TG)-induced Ca^2+^ mobilisation was analysed by exposing HCT116 cells to 0.05% DMSO and to a dilution series of mycolactone from 3.9 nm/ml to 125 ng/ml for 6 h. Cytosolic Ca^2+^ ([Ca^2+^]_cyt_) was imaged with FURA-2 (**A**). The area under the curve (AUC) was calculated from TG-induced Ca^2+^ transients (**B**). The number of cells is given within the graph bars in **B**. Data is presented as means ± SEM; *** *P* < 0.001.

**Figure 3.**
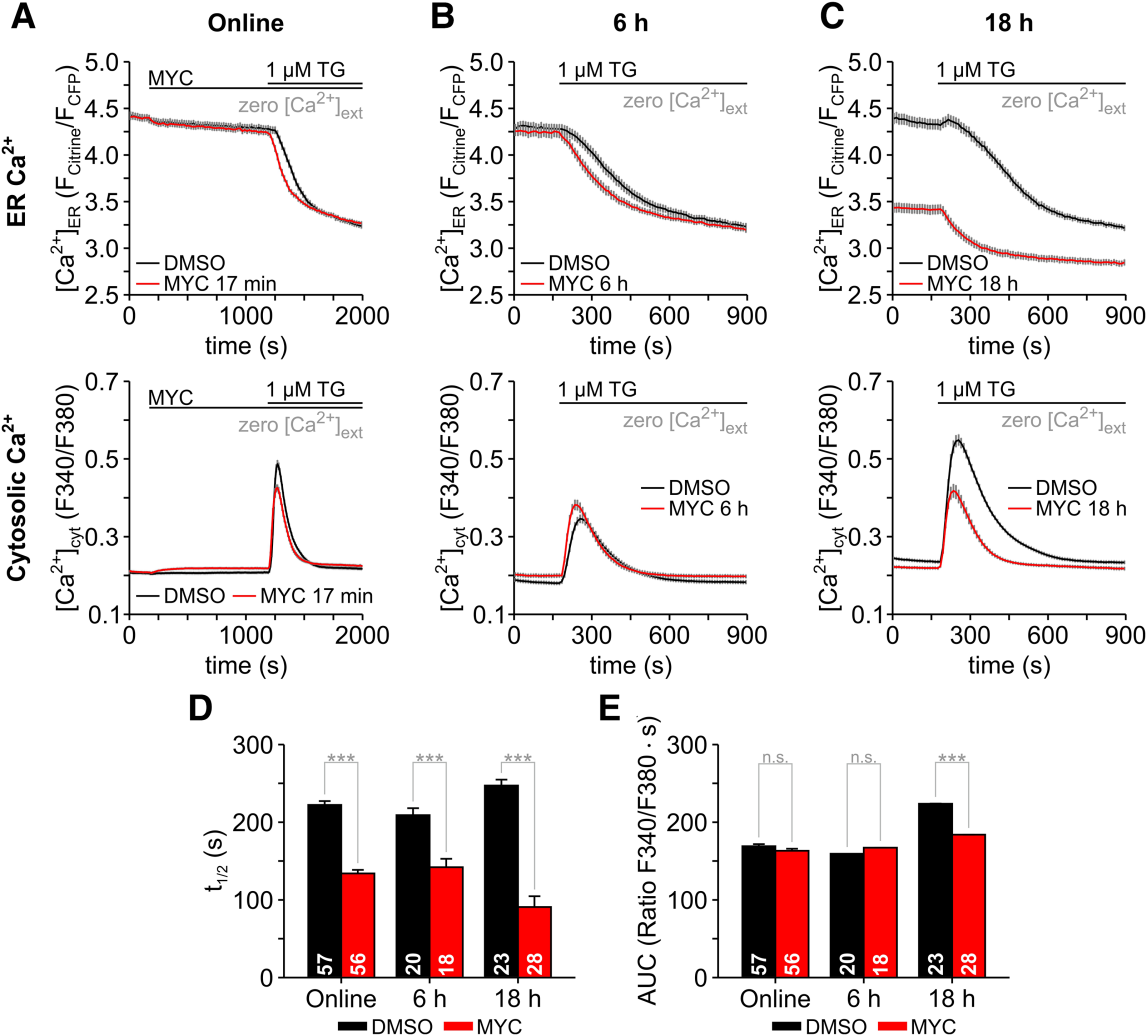
Mycolactone enhances the Ca^2+^ leak and induces Ca^2+^ depletion in the ER. Changes of the Ca^2+^ concentration in cytosol ([Ca^2+^]_cyt_) and ER ([Ca^2+^]_ER_) were imaged with FURA-2 and with the FRET-based D1ER sensor, respectively. Changes in cytosolic and ER Ca^2+^ are expressed as F340/F380 and F_Citrine_/F_CFP_ ratios. HEK-293 cells expressing D1ER (D1ER-HEK) were exposed to mycolactone (MYC) ‘online' for 17 min while the Ca^2+^ imaging experiment was running (**A**, MYC 17 min, 125 ng/ml mycolactone). Subsequently, thapsigargin (1 µM TG) was applied to unmask the Ca^2+^ leak form ER. In further experiments, D1ER-HEK cells were treated with mycolactone for 6 and 18 h before Ca^2+^ imaging (**B**, MYC 6 h, 100 ng/ml mycolactone; **C**, MYC 18 h, 100 ng/ml mycolactone). DMSO 0.025% was used as a control. *Upper panels* in **A**, **B** and **C** depict changes of ER Ca^2+^ content; *lower panels* show the corresponding transients in cytosolic Ca^2+^. Mycolactone and thapsigargin application protocols are depicted above graphs. The speed of TG-induced Ca^2+^ depletion in ER was measured as the time that D1ER ratios needed to decrease to 50% (**D**, *t*_1/2_) and AUC (**E**) was calculated for the Ca^2+^ transients in cytosol. The number of cells is given within the graph bars in **D** and **E**. Data is presented as means ± SEM; n.s., non-significant; *** *P* < 0.001.

**Figure 4.**
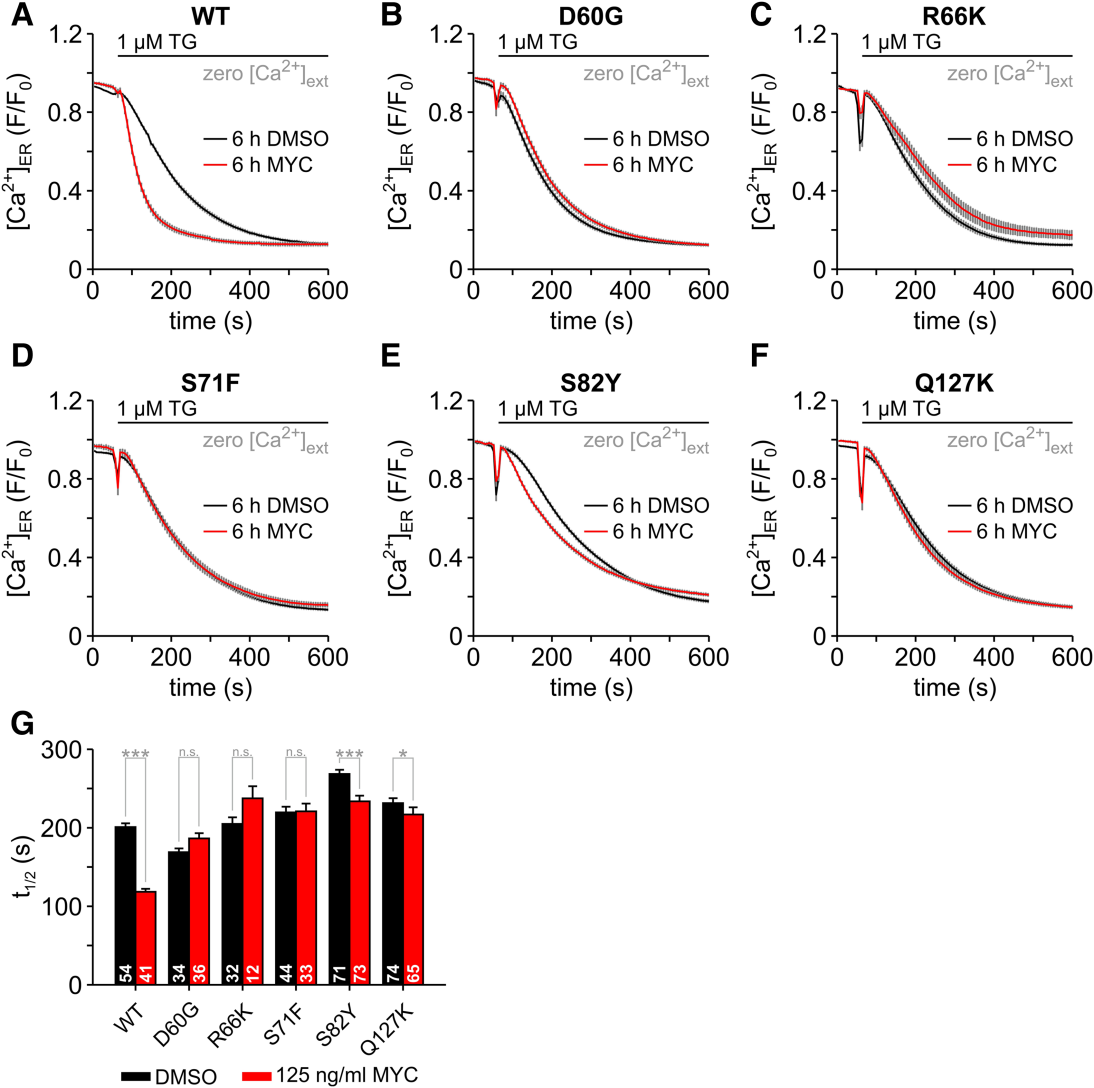
Mycolactone-resistant mutations in Sec61α abolish the effects of mycolactone on the Ca^2+^ leak from ER. The effects of mycolactone (MYC) on [Ca^2+^]_ER_ were analysed in wild-type HCT116 cells (**A**, WT) and in HCT116 cells containing point mutations in Sec61α (**B**, D60G; **C**, R66K; **D**, S71F; **E**, S82Y; **F**, Q127K). To image [Ca^2+^]_ER_, cells were transfected with the ER Ca^2+^ sensor ER-GCaMP6-150. Changes in [Ca^2+^]_ER_ are given as normalised fluorescence (*F*/*F*_0_). Cells were treated with 0.05% DMSO or 125 ng/ml mycolactone for 6 h before Ca^2+^ imaging. Thapsigargin (1 µM TG) was used to induce ER Ca^2+^ depletion. Accordingly, the speed in the decrease in [Ca^2+^]_ER_ is a measure of the Ca^2+^ leak from ER and was calculated as the time to 50% decrease in the normalised fluorescence *F*/*F*_0_ (**G**, *t*_1/2_). The number of cells is given within the graph bars in **E**. Data is presented as means ± SEM; * *P* < 0.05; *** *P* < 0.001.

Single cell data has been obtained in independent Ca^2+^ imaging recordings with 3–6 cover slips per experimental setting. Data was pooled and analysed with Excel 2010, Prism5 and Sigma Plot 10.0. The total number of analysed cells in each experimental setting is given in Figures 1–4 and Supplementary Figures S3, S4. Statistical significance of the Ca^2+^ imaging data was assessed with the two sample Kolmogorov–Smirnov test. Statistical significance is given as n.s., non-significant; * *P *< 0.05; ** *P *< 0.01 and *** *P *< 0.001.

## Results

### Impact of mycolactone on the cellular Ca^2+^ homeostasis

Mycolactone has previously been shown to target the α subunit of Sec61 complexes in the ER membrane [12,16]. Biochemical evidence from translocation assays support its inhibition of Sec61α at a step after the engagement of ribosomes [18], raising the possibility that one aspect of the cellular action of mycolactone is the disruption of the Ca^2+^ homeostasis due to its interaction with Sec61α within the ER membrane. To explore this possibility, we analysed the Ca^2+^ homeostatic mechanisms in cells treated with mycolactone. We imaged the cytosolic Ca^2+^ concentration ([Ca^2+^]_cyt_) with FURA-2 as well as the Ca^2+^ concentration in ER ([Ca^2+^]_ER_) with the genetically encoded sensors D1ER and ER-GCaMP6-150 [34,36].

Since [Ca^2+^]_ER_ is maintained ∼100–800 µM by a dynamic equilibrium between the leak of Ca^2+^ ions from the ER and the action of SERCA pumping Ca^2+^ back into the ER, as well as by the Ca^2+^ buffering of ER resident proteins [46,47], the SERCA-inhibitor thapsigargin (TG) unmasks the Ca^2+^ leak producing a transient increase in [Ca^2+^]_cyt_. Typically, TG-induced Ca^2+^ transients are characterised by a rapid onset and short duration in most mammalian cells, provided that the entry of Ca^2+^ ions into the cell has been suppressed by removing extracellular Ca^2+^[48]. Figure 1A–C illustrates the effects of 125 ng/ml mycolactone on the Ca^2+^ mobilisation induced by TG in HCT116 cells in the absence of external Ca^2+^. When Ca^2+^ transients of mycolactone-treated cells were compared with the DMSO controls, we observed that mycolactone reduced both the duration and the peak amplitude of the cytosolic Ca^2+^ transients after 1, 6 and 18 h of exposure (Figure 1A–C). Such effects are best quantified as the area under the curve (AUC), which is calculated by integration of the Ca^2+^ transients. Figure 1D shows that mycolactone significantly reduced the AUC of TG-induced Ca^2+^ transients, indicating that the amount of Ca^2+^ mobilised by TG from ER is lower in mycolactone-treated cells. Similar effects of mycolactone on cytosolic Ca^2+^ transients were also observed with macrophage RAW 264.7 cells (Supplementary Figure S3). Since ER Ca^2+^ depletion as well as increased Ca^2+^ leak from ER can explain the attenuation of TG-induced Ca^2+^ transients by mycolactone, we next used ionomycin (IONO) in order to estimate the total amount of Ca^2+^ that can be mobilised in HCT116 cells. As shown in Figure. 1E, the IONO-induced Ca^2+^ transients had a reduced amplitude and were shorter in mycolactone-treated cells than in DMSO controls. The AUC analysis revealed that HCT116 cells lost ∼2/3 of the mobilisable Ca^2+^ after 6 h treatment with 125 ng/ml mycolactone (Figure 1F). Thus, mycolactone likely compromises the Ca^2+^ homeostasis in a way that reduces the Ca^2+^ storage levels and attenuates Ca^2+^ signalling in HCT116 cells. These observations cannot be explained by cytotoxicity since the viability of these cells after 18 h mycolactone exposure was similar to DMSO control cells (Figure 1G).

In HCT116 cells, less than 10 ng/ml mycolactone is sufficient to maximally reduce cell viability [15] after 5 days exposure. Since we used 125 ng/ml mycolactone in previous experiments (Figure 1), the question arises as whether low concentrations of mycolactone also induce attenuation of TG-induced Ca^2+^ transients. Therefore, we next exposed HCT116 cells to a dilution series between 3.9 ng/ml and 125 ng/ml mycolactone for 6 h. Thereafter, cells were exposed to TG to induce Ca^2+^ mobilisation from ER. As shown in Figure 2A, 3.9 ng/ml mycolactone shortened the Ca^2+^ transients without major effect on the amplitude. Higher concentrations of mycolactone shortened the TG-induced Ca^2+^ transients further and these transients displayed noticeably smaller amplitudes, with the consequence that the AUC of TG-induced Ca^2+^ transients displayed a dose-dependent inhibition by mycolactone (Figure 2B).

### Mycolactone enhances the Ca^2+^ leak from ER

We found that the total amount of mobilisable Ca^2+^ in HCT116 cells is reduced by mycolactone (Figure 1E,F), suggesting that the ER Ca^2+^ content may be compromised in mycolactone-treated cells. We hypothesised that an increased Ca^2+^ leak that leads to ER Ca^2+^ depletion during the exposure to mycolactone likely contributes to the attenuation of Ca^2+^ transients that were observed in HCT116 cells (Figure 1A–C), because the amplitude and duration of TG-induced Ca^2+^ transients are determined by Ca^2+^ content of the ER as well as by the Ca^2+^ leak from ER. To test this hypothesis, we therefore imaged the changes in [Ca^2+^]_ER_ and [Ca^2+^]_cyt_ in the same cells (Figure 3). [Ca^2+^]_ER_ was imaged using the FRET-based ER Ca^2+^ sensor D1ER [35], which was stably expressed in HEK-293 cells (D1ER-HEK) [34]. D1ER-HEK cells were additionally loaded with FURA-2 to image [Ca^2+^]_cyt_. In the first series of experiments, we applied mycolactone to D1ER-HEK cells for 17 min ‘online' in order to visualise possible immediate mycolactone effects (Figure 3A). Surprisingly, while the cytosolic Ca^2+^ levels slightly increased during the exposure to mycolactone, ER Ca^2+^ was not affected by mycolactone. At the end of the exposure to mycolactone, the TG-application induced a depletion of ER Ca^2+^ that reflects the unmasking of the Ca^2+^ leak from ER, as previously reported [34]. As shown in Figure 3A (*upper panel*), the time course of ER Ca^2+^ depletion was faster in D1ER-HEK cells treated with mycolactone when compared with DMSO controls. Analysis of the time to 50% decay in ER Ca^2+^ (*t*_1/2_) revealed a 1.6 times faster decay of [Ca^2+^]_ER_ in cells treated with mycolactone for 17 min (Figure 3D). This fast Ca^2+^ depletion is indicative of a mycolactone-induced enhancement of the Ca^2+^ leak from ER within minutes of exposure. By prolonging the exposures to 6 and 18 h, the effects of mycolactone became more pronounced (Figure 3B,C, *upper panels*) and t_1/2_ became 2.7 faster at 18 h exposure (Figure 3D). Additionally, we observed that the basal ER Ca^2+^ levels decreased after 18 h mycolactone treatment (Figure 3C, *upper panels*), indicating that mycolactone enhances the Ca^2+^ leak from ER, which in the long term produces a continuous Ca^2+^ depletion in the ER. The corresponding effects of mycolactone on the cytosolic Ca^2+^ transients were less pronounced in the ‘online' and 6 h experiments but the duration and peak amplitude of the transients were strongly reduced after 18 h mycolactone treatment (Figure 3A–C, *lower panels*). Accordingly, the AUC of the Ca^2+^ transients was only significantly reduced after the 18 h mycolactone treatment (Figure 3E). The most parsimonious implication of this observation is that mycolactone effects are best detectable at the level of cytosolic Ca^2+^ transients when the enhanced Ca^2+^ leak has produced a considerable ER Ca^2+^ depletion, as illustrated by the 18 h experiment (Figure 3C, *lower panel*). However, the enhancement of Ca^2+^ leak induced by mycolactone was detectable as accelerated ER Ca^2+^ depletion as early as 17 min after treatment (Figure 3A, *upper panel*).

### Sec61 complexes are involved in the mycolactone effects on ER Ca^2+^ leak

Since mycolactone targets the α subunit of Sec61 complexes in the ER membrane [12,16], altering its conformation [16,18], and Sec61 complexes form a Ca^2+^ leak pathway from ER [49], a distinct possibility is that mycolactone stabilises a conformation, in which Sec61 complexes are permeable to Ca^2+^. To test this hypothesis, we took advantage of a series of mycolactone-resistant HCT116 cell clones that have heterozygous non-synonymous mutations within the *SEC61A1* gene encoding Sec61α, which have been shown to reduce mycolactone binding to the pore-forming major subunit [15,16]. For our Ca^2+^ imaging experiments, we selected the cell line expressing Sec61α D60G, which is located in the cytosolic loop (CL) 1 of Sec61α, as well as the cell lines expressing Sec61α S82Y and Q127K, which are located in the transmembrane segments (TM) 2 and 3, respectively, and the mutations R66K and S71F which are located in the transition between CL1 and TM2. Using the ER Ca^2+^ sensor ER-GCaMP6-150 [36], we have visualised the TG-induced depletion of ER Ca^2+^ as a direct surrogate of Ca^2+^ leak in these cell lines (Figure 4). As expected, ER-GCaMP6-150 detected an exponential Ca^2+^ depletion in wild-type (WT) HCT116 cells (Figure 4A), as well as in all clones containing Sec61α mutants (Figure 4B–F). Notably, the rate of Ca^2+^ depletion was different in each clone as revealed by the t_1/2_ values (Figure 4G), particularly for D60G and S82Y. This suggests that the mutations themselves may impact the structure and function of Sec61 translocons, as expected as they are *prl* mutants.

In WT HCT116 cells mycolactone reduced the *t*_1/2_ by ∼45% (Figure 4A,G), in line with the observation using the ER Ca^2+^ sensor D1ER (Figure 3D). However, the effects of mycolactone on cells expressing the Sec61α mutants varied slightly according to the mutation. For instance, mycolactone no longer caused any change to the t_1/2_ with the Sec61α S71F mutant (Figure 4D,G). Although not statistically significant, longer *t*_1/2_ values were observed in the Sec61α D60G and Sec61α R66K mutants (Figure 4B,C,G). Compared with the respective DMSO controls, significantly shorter *t*_1/2_ values were detected in the Sec61α mutants S82Y and Q127K (Figure 4E–G), although these mycolactone effects were always less pronounced than the nearly 45% reduction in *t*_1/2_ in WT HCT116 cells. In the case of the Sec61α S82Y mutant, mycolactone reduced *t*_1/2_ by ∼12% (Figure 4E,G), a much milder effect. All in all, the direct analysis of the TG-induced ER Ca^2+^ depletion provided evidence that mycolactone no longer enhances Ca^2+^ leak from ER in HCT116 cells expressing the mycolactone resistance mutations in Sec61α (Figure 4). Hence, our results strongly support the suggestion that Ca^2+^ leak from ER in the presence of mycolactone is mediated by Sec61α. Supporting this suggestion, siRNA knockdown of *SEC61A1* in HeLa cells abolished the enhancing effects of mycolactone on Sec61α-mediated Ca^2+^ leak (data not shown). Furthermore, the absent or weakened Ca^2+^ leak from the ER could be a contributing factor to the increased mycolactone resistance of these cell lines in cell survival assays [15,16].

### Molecular docking

**Figure 5.**
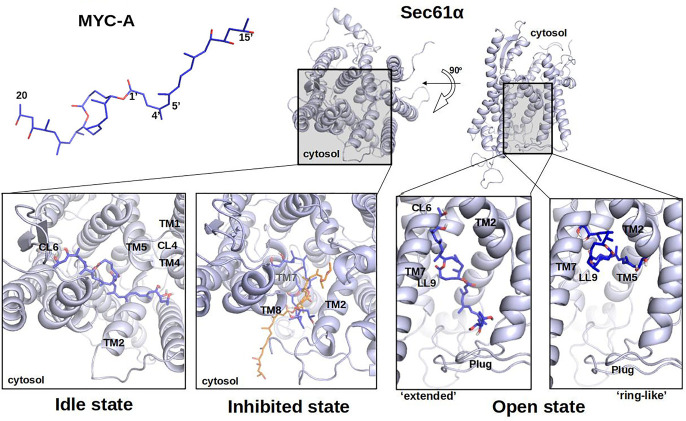
Most favourable binding poses of mycolactone A with Sec61α. Mycolactone A (MYC-A, *top-left*) was docked to homology models of human WT Sec61α. Overviews depict Sec61α seen from the cytosol and from the plane of the membrane with the cytosolic side upward (Sec61α, *top-right*). The most favourable binding poses of mycolactone A in the *idle* (*lower-left*), *mycolactone-inhibited* (*lower-middle*) and *open* (*lower-right*) state-homology models are shown. The mycolactone conformation from cryo-EM structure PDB 6Z3T (*orange*) is super imposed on the docking result for the *inhibited* state . For the *open* state, two different conformations are shown; ‘extended' and ‘ring-like'. TM, transmembrane helix; CL, cytoplasmic loop.

**Figure 6.**
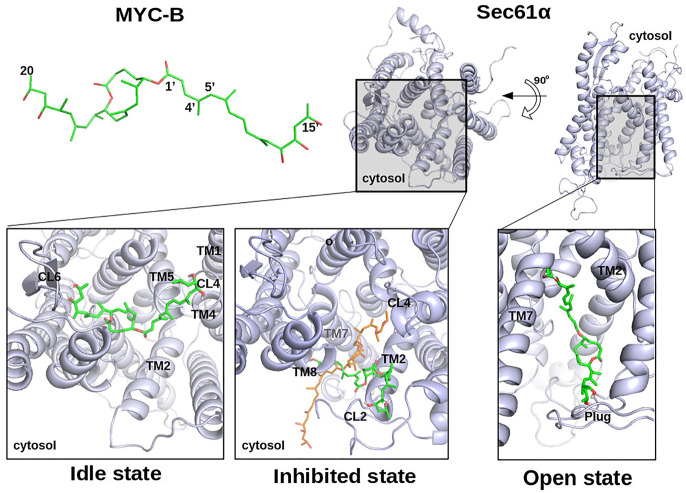
Most favourable binding poses of mycolactone B with Sec61α. Docking of mycolactone B (MYC-B, *top left*) was performed on homology models of human WT Sec61α. The overviews show Sec61α from the cytosol and from the membrane side plane (Sec61α, *top-right*). The most favourable binding poses of mycolactone B in the *idle* (lower-left), *inhibited* (lower-middle) and *open* (lower-right) state- homology models are shown. The mycolactone conformation from cryo-EM structure PDB 6Z3T (*orange*) is super imposed on the docking result for the *inhibited* state. TM, transmembrane helix; CL, cytoplasmic loop.

For our homology model of the *idle* conformation of human Sec61α within ribosome-Sec61 complexes, docking yielded favourable low-energy conformations where mycolactone A and B interacted with Sec61α with a binding energy of −7.25 ± 0.8 kcal/mol and −8.17 ± 0.5 kcal/mol (on average), respectively. Both mycolactone isomers were placed near the cytosolic entrance of the *idle* WT Sec61α pore (see Figures 5 and 6, *bottom left panels*) and mainly occupied the volume between cytosolic loops 6 and 4 (CL6 and CL4). The long acyl tails (Southern tail) of both mycolactones face towards the CL4 region. The docking results suggest that the Northern acyl side chain of both mycolactone A and B could potentially form hydrogen bonds with the CL6 region because residues from the region Pro280–Ser287 (CL6–TM7) were predicted to strongly interact with both isomers of mycolactone.

For the mycolactone-inhibited conformation, the docking results suggest that mycolactone occupies the groove between TM8, TM2 and TM7 helices (Figures 5 and 6, *bottom middle panels*). The docking position of the lactone core of mycolactone compares well to the orientation of mycolactone determined in the EM structure [16]. In contrast with the EM structure, the longer acyl side chain (southern chain) of mycolactone is oriented towards the lateral gate (Figures 5 and 6, *bottom middle panels*), possibly due to the absence of lipids in the docking analysis. These docking results suggest that both mycolactones A and B can interact with this conformation of Sec61α with similar binding energies of −8.21 ± 1.1 and −8.67 ± 0.6 kcal/mol, respectively. Residues from the region Leu89–Lys98 (TM2) strongly interact with both isomers of mycolactone. The most favourable final binding poses of mycolactone A and B are displayed in Figures 5 and 6, respectively.

For the *open* conformation of Sec61α, the docking results placed mycolactone inside the channel pore that would normally be the translocation path of substrate peptides. Mycolactone A was placed here in two main arrangements that we refer to as ‘ring-like’ or ‘extended’ conformations (Figure 5, *bottom right panel*). The ‘extended’ conformation was slightly more favourable with a binding energy of −9.02 ± 0.5 kcal/mol than the ‘ring-like’ conformation (−8.21 ± 0.6 kcal/mol). In the ‘extended’ conformation, the acyl side chains of mycolactone A often formed hydrogen bonds with residues in CL6, lumenal loop (LL) 9 and the plug region. In the ‘ring-like’ conformation, mycolactone A interacted with the N-terminal region of TM2, LL9 and TM5. mycolactone B also adopted the ‘extended’ conformation (see Figure 6) inside the channel pore and the ligand pose looked like a ‘bridge’ between the plug region and the loop CL6 similar to mycolactone A. The acyl side chains of mycolactone B often interacted with residues of CL6, LL9, plug and TM5. The lowest binding energy of mycolactone B was −8.78 ± 0.6 kcal/mol.

### Ca^2+^ leak due to mycolactone is not due to ribosome dissociation from translocons

**Figure 7.**
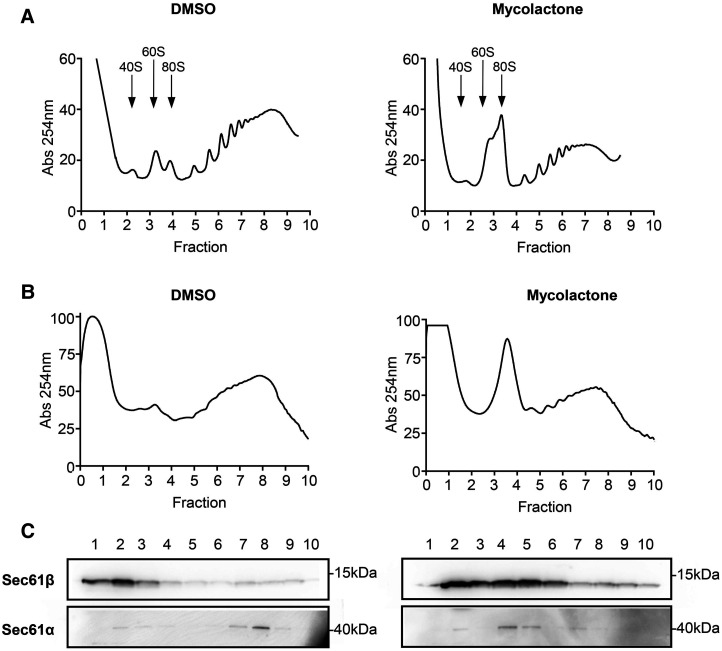
Mycolactone promotes the disruption of polysomes. Typical polysome profile of whole cell triton-x-100 extracts from RAW 264.7 cells treated with DMSO or 31.25 ng/ml mycolactone (**A**) and of membrane associated polysomes from RAW 264.7 cells treated with DMSO or 31.25 ng/ml mycolactone for 4 h (**B**). Membrane-associated polysomes were isolated from semi-permeabilised cell extracts solubilised with 2% digitonin and separated on a 10–60% sucrose gradient as described in Material and Methods (**B**). Proteins within column fractions, which correspond to those in the polysome profiles directly above, were acetone-precipitated and subject to SDS page and immunoblotting with anti-Sec61α and anti-Sec61β antibodies (**C**). Figure representative of duplicate assays.

Our data suggest that mycolactone causes Ca^2+^ leak predominantly by altering the structure of Sec61α within the Sec61 translocon. However, other molecules that associate with the translocon could also cause increased Ca^2+^ leak if their function was altered. For instance, BiP (HSPA5) is known to bind to Sec61α in the region of ER lumenal loop 7 and depletion increases Ca^2+^ leak [26]. *A priori*, however, while BiP is a Sec61 substrate that should be mycolactone sensitive, it is known not to be reduced for at least 16–24 h following exposure in a wide range of different cell types [13,14, Hall, Hsieh and Simmonds et al., unpublished]. Alternatively, ribosome disassembly and subsequent loss of polysomes, for instance by puromycin [50], also increases the Sec61-mediated Ca^2+^ leak from the ER [51]. Previous studies have shown that mycolactone reduces the abundance of polysomes and increases that of the 60S ribosomal subunit peak in the sub-polysomes in 1% triton cell extracts, where cytosolic ribosomes predominate [11], with typical profiles shown in Figure 7A (DMSO*, left panel*; mycolactone, *right panel*). To investigate the effect of mycolactone on ribosome association with mRNA at the ER, we analysed the membrane-associated polysome profile of cells exposed to mycolactone using digitonin extracts. In DMSO control cells, although the yield was lower, the profile was similar to that of the cytosolic fraction (Figure 7A,B, *left panel*). Following 4 h mycolactone exposure, the polysomal peak was reduced, with an increase in the 80S peak (Figure 7B, *right panel*). In addition, immunoblotting of the polysome gradient fractions showed a shift of Sec61α from the polysomal fraction in control cells to the 80S fraction in mycolactone-treated cells (Figure 7C; compare DMSO, *left panel*, with mycolactone, *right panel*). Sec61β is expressed in excess over Sec61α in cells, and is therefore found widely in ER membranes, not just in association with Sec61α [49]. Hence it can be also seen accumulating in the 80S fractions with Sec61α in mycolactone treated cells, indicating the presence of intact translocons. This data suggests that the addition of mycolactone results in the disassembly of polyribosomes leaving isolated ribosome-Sec61 complexes in the ER membrane. Furthermore, it confirms previous data from extracted canine microsomes that mycolactone appears not to displace ribosomes from the ER [16,18].

## Discussion

In this work, we present novel data from Ca^2+^ imaging and show how application of the Sec61α inhibitor mycolactone affects Ca^2+^ leak through Sec61 translocons. Also, we present results from molecular docking aimed at identifying putative binding modes of mycolactone to various conformational states of the pore-forming subunit Sec61α. We will start this discussion by summarising the existing experimental data on how mycolactone affects Sec61α function. Then, we will connect this with the available structural data and docking results and, finally, suggest an integrated mechanistic model for mycolactone action on Sec61 complexes.

It has been known for some time that Sec61 complexes can facilitate the leak of Ca^2+^ ions from the ER to the cytosol [51], which are normally pumped back into the ER by the SERCA pump. Application of the SERCA pump inhibitor TG prevents this and leads to emptying of the ER Ca^2+^ due to the Ca^2+^ leak. This is followed by partitioning of Ca^2+^ to other intracellular compartments or clearance from cells. Accordingly, we observed an ER Ca^2+^ depletion that followed an exponential time course after TG application to D1ER-HEK, where the Ca^2+^ leak determines the speed of Ca^2+^ depletion (Figure 3A–C, *upper panels*). Mycolactone increased the speed of ER Ca^2+^ depletion with treatments as short as 17 min and, therefore, we suggest that the enhancement of Ca^2+^ leak from ER is one of the earliest events within the cellular action of mycolactone (Figure 3A, *upper panel*). The TG-induced cytosolic Ca^2+^ transients depicted in Figure 3A–C (*lower panels*) reflected both the speed of Ca^2+^ depletion as well as the absolute amount of Ca^2+^ contained in the ER. In general, when the Ca^2+^ leak increases, the cytosolic Ca^2+^ transients became shorter because the ER is quickly depleted of Ca^2+^. Additionally, the amplitudes of Ca^2+^ transients decrease when the ER Ca^2+^ content is reduced by a sustained high Ca^2+^ leak. In line with this suggestion, TG-induced cytosolic Ca^2+^ transients of small amplitude and short duration were detected in D1ER-HEK cells only when the Ca^2+^ leak has considerably depleted the ER of Ca^2+^ during 18 h exposures to mycolactone (Figure 3C, *lower panel*). Similarly, small and short TG-induced cytosolic Ca^2+^ transients were observed after exposures of HCT116 and RAW 264.7 cells to mycolactone for more than 1 h (Figure 1 and Supplementary Figure S3). Furthermore, we found that the effects of mycolactone on Ca^2+^ leak increase dose-dependently (Figure 3), presumably as the available Sec61 translocons become occupied with mycolactone, since their stoichiometry is approximated 1:1 [16].

**Figure 8.**
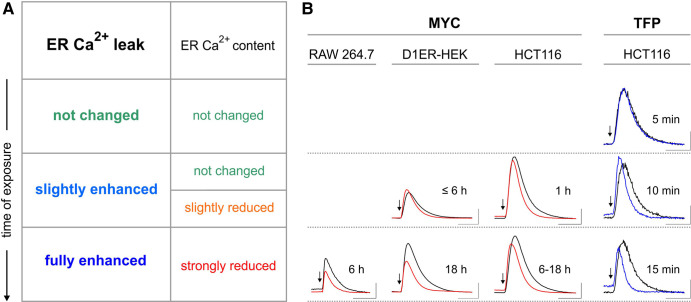
Mode of mycolactone action on the Ca^2+^ homeostasis. Upon application *in cellula*, mycolactone (MYC) enhances gradually the Ca^2+^ leak from ER and, as a consequence, there is a steady loss of Ca^2+^, which in turn reduces the ER Ca^2+^ content with respect to controls (**A**). Since the cytosolic TG-induced Ca^2+^ are shaped by the ER Ca^2+^ leak and ER Ca^2+^ content, mycolactone reduces the amplitude and duration of these Ca^2+^ transients in a time dependent manner. Shown are representative examples of TG-induced Ca^2+^ transients recorded in HCT116, D1ER-HEK and RAW 264.7 cells exposed to mycoalctone (**B**, MYC). The exposure time is indicated next to each Ca^2+^ transient (*black*, DMSO controls; *red*, mycolactone-treated cells). The time point of TG application is marked by an arrow. The dwindling of TG-induced Ca^2+^ transients upon application of mycolactone is recapitulated in HCT116 cells treated with the reference Ca^2+^ leak enhancer trifluoperazine (**B**, TFP. *Black*, DMSO controls; *blue*, TFP-treated cells). The Ca^2+^ transients in **B** are depicted as Δ[Ca^2+^]_cyt_ or ΔF340/F380. Scales: RAW 264.7, 0.2 ratio, 200 s; D1ER-HEK, 0.15 ratio, 200 s; HCT116, 0.2 µM, 200 s. Same experiments as in Figures 1A–C, 3A–C (*lower panels*) and Supplementary Figures S3 and S4.

Thus, our data collectively suggests that the effects of mycolactone on the Ca^2+^ homeostasis build from the enhancement of ER Ca^2+^ leak to the attenuation of cytosolic Ca^2+^ signalling (Figure 8). Because mycolactone crosses cell membranes by passive diffusion and accumulates in the cell in a time-dependent manner [3,32], it causes a gradual increase in the ER Ca^2+^ leak and a slow decrease in ER Ca^2+^ content upon application *in cellula* (Figure 8A). The corresponding dwindling of TG-induced Ca^2+^ transient was observed in RAW 264.7, D1ER-HEK and HCT116 cells with at least 1 h exposure to mycolactone and appears to be a general feature of the mycolactone action (Figure 8B). The time-scale of effects is, however, quite different from those reported for other modulators of Sec61-mediated Ca^2+^ leak such as eeyarestatin, which is predicted to bind Sec61 complexes in the *open* state [34,49]. For comparison, therefore, we exposed HCT116 cells to the calmodulin inhibitor trifluoperazine (TFP, Supplementary Figure S4), which is an enhancer of Sec61 mediated Ca^2+^ leak [52]. As shown in Figure 8B, the time-dependent dwindling of TG-induced Ca^2+^ transients was recapitulated by the exposure to TFP, albeit much faster with a time scale of minutes. We presume that this difference is due to the different modes of action, where TFP inhibits calmodulin, which normally limits the Sec61-mediated Ca^2+^ leak [52], and mycolactone promotes a putative *intermediate* state of Sec61α [16] with corresponding effects on ribosome recycling (see below). TFP may also enter cells faster than mycolactone. Interestingly, but perhaps unsurprisingly, there is a reasonable correlation between the slow action on Ca^2+^ leak and the kinetics of toxicity, since mycolactone-exposed cells take 4–5 days to die [53]. Thus, the comparison of mycolactone and TFP suggest that the dwindling of TG-induced Ca^2+^ transients in cells exposed to mycolactone follows a general mechanism based on the enhancement of Ca^2+^ leak from ER (Figure 8B).

Further analyses of the ER Ca^2+^ depletion in HCT116 cells carrying mycolactone-resistant Sec61α mutations enabled us to pin down the role of Sec61 translocons in the effects of mycolactone on the Ca^2+^ leak from ER (Figure 4). We observed no statistically significant effects of mycolactone with the Sec61α mutants D60G, R66K and S71F, indicating that mycolactone has no effect on the Ca^2+^ leak supported by these Sec61α mutants. Mycolactone induced a slight increase in Ca^2+^ leak only in cells expressing the mutants S82Y and Q127K, but these effects were by far less pronounced than that in wild-type cells. This demonstrates that the effect of mycolactone on Ca^2+^ leak is extremely likely to be related to its interaction with Sec61α rather than its other reported targets, or a general disturbance of phospholipid bilayer integrity. The effects of mycolatone on the Ca^2+^ leak mediated by the S82Y and Q127K mutants may reflect differences in the effect of the mutations on the structure and function of the Sec61 translocon. This is not unexpected, since most have been previously associated with the *prl* phenotype, which alters the efficiency of translocation depending on the nature of the signal peptide [16]. Further Ca^2+^ imaging experiments are required to characterise the kinetics of the Ca^2+^ leak mediated by mycolactone-resistant Sec61 mutants.

McKenna et al.[18] found that ribosomes still bind to the Sec61 complex in the presence of mycolactone but observed discrete changes in the architecture of the ribosome-nascent chain (RNC)-Sec61 interaction, as evidenced by cross-linking of the nascent chain to Sec61 subunits. Additionally, mycolactone treatment altered the trypsin sensitivity of CL6 and CL8 (implicated in ribosome binding) of Sec61α. On this basis, this biochemical analysis favoured a model where mycolactone perturbs the interaction between signal peptide and the ribosome–Sec61 complex that is necessary for co-translational translocation to progress. The recent analysis of the mycolactone-bound translocon shows that the conformation of the translocon is no longer similar to that of the *idle* translocon, with Sec61α instead adopting a position that is wedged open at the lateral gate [16]. The lactone ring of mycolactone binds near the lateral gate and stabilises it in a partially open conformation.

Molecular re-docking of mycolactone into a homology model of the EM structure of the mycolactone-bound state confirms the plausible position of the lactone ring identified by Gérard et al. [16]. Previously, Aydin et al. [54] characterised the orientational preference of mycolactone in a plain DPPC bilayer by molecular dynamics simulations. They reported that the highly hydrophobic lactone ring prefers to be buried among the hydrophobic lipid tails of the phospholipid bilayer, whereas the hydroxyl groups that are found on the side chains either tend to stretch out to the hydrophilic lipid headgroups or interact with water molecules. Hence, the more extended conformation of mycolactone reported by Gérard et al. [16] appears more plausible than the compact conformation identified in the docking run. Molecular docking of mycolactone to the *idle* state also revealed a novel potential binding site on the surface of Sec61α. Molecular docking of mycolactone to the open state identified further putative binding positions in the channel pore. However, noting the just mentioned preference of mycolactone's lactone ring and its Northern and Southern chains for a patterned, mixed hydrophobic/hydrophilic environment [54], these docking positions in the water-filled hydrophilic pore of the open state appear less likely. Also, there is so far no experimental evidence supporting such binding modes.

Similar to Gérard et al. [16], these docking runs position mycolactone with the core and Northern chain within the translocon and the Southern chain extending from the translocon into the ER membrane. It has been known for some time that the Southern chain of mycolactone is important for cytotoxic and/or cytokine suppressing activity of mycolactone [3]. Since forward genetic screens have unambiguously linked these readouts to Sec61α [12,15,16], this data can be used as a retrospective surrogate for Sec61 complex inhibition. Here, naturally occurring mycolactones with differing specific activities are invariant in the Northern chain and lactone core, but vary in the Southern chain methylation, hydroxylation and/or length. Furthermore, wide-ranging structure-function studies using synthetic variants of mycolactone [55] have revealed that variations in the Northern chain are generally well-tolerated, whereas the Southern chain is much more sensitive to even small perturbations in structure. After addition of even bulky functional groups to the Northern chain at C14 or C20 mycolactone retains cytotoxic activity. On the other hand, truncation of the Southern chain to C6′ or C2′, or completely removing it results in a molecule with much reduced activity. A synthetic mycolactone produced by Scherr et al. [56], with no hydroxylation of the Southern chain, was found to retain minimal cytotoxic activity. More specifically to translocation, structural variants of mycolactone lacking the Northern chain can still compete with cotransin (CT7) for Sec61α binding, whereas those lacking the Southern chain or both chains cannot [12]. These data strongly suggest that the lactone core and Northern chain interacts directly with TM2 of the translocon, explaining the evolutionary conservation of these elements of the structure. On the other hand, we propose that the Southern chain has essential interactions with the lipid bilayer and ascribe the activity differences in Southern chain variants to differing abilities to interact with the lipid bilayer. Interactions of the tail groups with phospholipid headgroups may serve to anchor the molecule, stabilising the inhibited conformation of the translocon.

**Figure 9.**
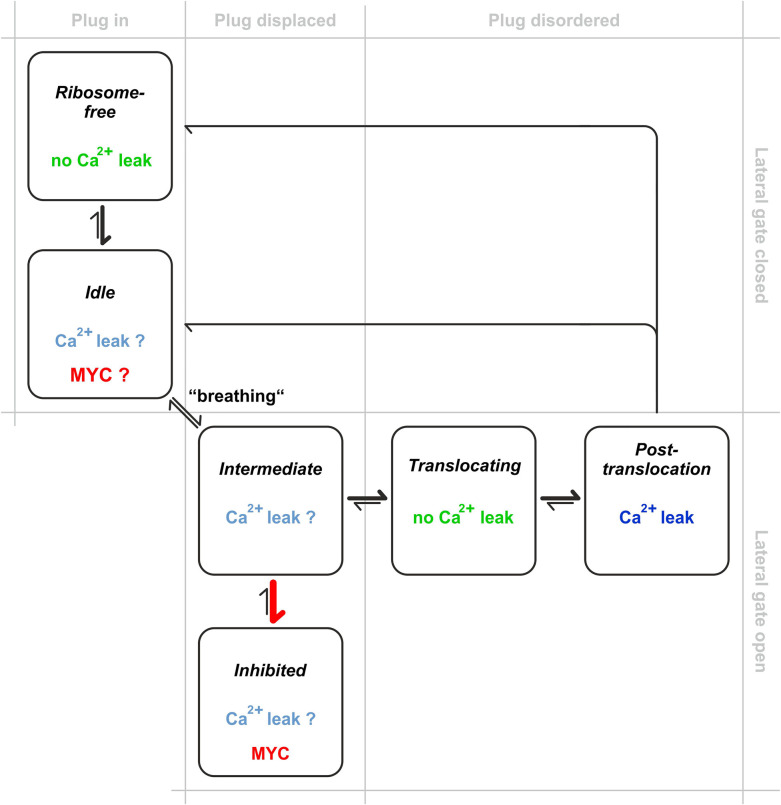
Model for mycolactone (MYC)-induced Ca^2+^ leak via Sec61 complexes. In normal translocation, *idle* translocons are engaged with ribosome nascent chain complexes, but co-translational translocation has not yet started. There is no evidence that *idle* translocons are Ca^2+^ permeable. Translocons ‘breathe' along the lateral gate, rapidly transitioning between the *idle* and *intermediate* states. We propose that the *intermediate* state may become transiently Ca^2+^ permeable due to its open structure and the partial displacement of the plug helix. Once the signal peptide of the nascent chain functionally engages with the lateral gate of Sec61α, the protein-conducting channel facilitates the translocation of the nascent protein by complete unfolding of the plug into a disordered state, but the *translocating* state is not permeable to Ca^2+^ due to the peptide filling the resultant channel. After translocation, Sec61 complexes transiently remain in this fully open *post-translocation* state, but without a nascent chain, allowing Ca^2+^ ions to permeate through the aqueous channel. The cycle is completed when the ribosomes de-attach and Sec61 complexes return to the *ribosome-fre*e or *idle* state, depending on whether the ribosome is released. Mycolactone traps translocons in an *inhibited* state, that resembles the *intermediate* state, but may also be able to bind Sec61α in the *idle* state. This locks the Sec61 translocon in a Ca^2+^-permeable conformation and an inefficient cycling through the other states, resulting in enhanced Ca^2+^ leak from the ER. Mycolactone causes an increase in membrane-associated 80S ribosomal monosomes. Hence, another contributing factor to the enhanced Ca^2+^ leak could be sustained dwell time in the *post-translocation* state, due to decreased efficiency of ribosome release.

The *idle* state represents a non-translocating ‘primed’ state of Sec61α bound to ribosomes with closed lateral gate and the plug in place. This state is represented by the structure reported by Voorhees et al. [21] and, so far, there is no evidence that Sec61 translocons support Ca^2+^ flux in this *idle* state [57]. After binding of the ribosome and shortly before engagement of the signal peptide within the lateral gate, we postulate that Sec61α rapidly transitions between this and an *intermediate* state, that closely resembles that of the yeast Sec62/Sec63-bound translocon required for post-translational translocation in that species. Bhadra et al. [58] recently characterised by molecular dynamics simulations how Sec63 modulates opening of the lateral gate of Sec61 in yeast. However, this transient structure has not yet been seen in the absence of mycolactone or other inhibitors [16]. It is plausible that the conformation of the *intermediate* state in Figure 9 may differ from that reported for the mycolactone-Sec61α complex, which has an open lateral gate, and the plug helix is intact but displaced. It is conceivable that ‘translocon breathing' could be associated with very transient states in which Ca^2+^ ions can leak out of the ER. If so, mycolactone's stabilisation of this state would clearly support the leak of Ca^2+^ ions observed even at doses where all translocons are expected to be saturated with mycolactone.

Considering the known three conformations of Sec61α (*idle*, *intermediate*, *open*), we postulate that mycolactone preferentially binds to, and stabilises, the *intermediate* conformation, which is structurally similar to the *inhibited* conformation. Translocon breathing suggests a mechanism for mycolactone's accessing of the translocon, as unbound mycolactone distributed in the ER membrane could penetrate the lateral gate during this step. This is in line with the structure-activity relationship data for mycolactone variants [56,59,60]. Alternatively, our docking analysis predicts that mycolactone could potentially bind on the cytosolic entrance of the Sec61α pore.

In the scheme of Figure 9, nascent chain translocation occurs after the plug has moved out of the pore (*translocating* state). Since the pore is entirely occupied by the translocating peptide, it is accepted that Sec61 complexes are not Ca^2+^ permeable in this state. When translocation is completed, the channel pore is briefly left open (*post-translocation* state) by the exiting peptide making this state Ca^2+^ permeable. Finally, the lateral gate and plug close again and the channel converts back to the *idle* state or *ribosome-free* state depending on whether the ribosome detaches from the translocon or not. While there is no evidence that Ca^2+^ ions permeate through Sec61 translocons in the *idle* state[57], functional studies in planar lipid bilayers demonstrated that Sec61 translocons support Ca^2+^ leak in the *post-translocation* state but not in the *translocating* and *ribosome-free* states[51,57,61].

Another mechanism that might conceivably influence Ca^2+^ leak from the ER, is the interaction of accessory factors with Sec61 complexes, such as chaperones or the ribosome. For example, reduced abundance of a constitutively expressed Sec61-dependent ER chaperone such as BiP might conceivably induce a similar phenotype. We have previously shown that such proteins are depleted at the turnover rate [62]. However, numerous proteomics studies have not shown any loss of BiP even at 16–24 h [13,14, Hall, Hsieh et al., unpublished], even though transcription is reduced [14]. These observations are consistent with its slow turnover rate [63], hence this is unlikely to be a major component of the explanation of Ca^2+^ leak, especially at earlier timepoints, including our on-line experiments and at 1 h exposure. However, the breakdown of ER-associated polysomes to a single 80S peak hints at effects of mycolactone on this stage of translocation that may also contribute to the Ca^2+^ leak. While translocation of secreted and Sec61α-dependent single-pass membrane proteins is blocked by mycolactone [64], many multi-pass membrane proteins are still being synthesised, not all of which are thought to be substrates of the ER membrane protein complex [65]. We found that monomeric ribosomes accumulate in cells treated with mycolactone, and these are still associated with Sec61α. This scenario could be explained by altered conformation of the RNC complex delaying ribosome release following translation termination. Alternatively, the loss of the polysomes may slow the transition between the *post-translocation* and *idle/ribosome-free* states, allowing more time for Ca^2+^ to leak out. Interestingly, this data is in line with a previously published study, which showed a similar effect in cells exposed to DTT or TG [66]. Like mycolactone, these compounds activate the integrated stress response pathways, and here the monosomal ribosomes continued to translate while associated with the membrane. Moreover, it agrees with our docking analysis that suggests that mycolactone can form hydrogen bonds with residues of CL6 on Sec61α, which interact directly with the ribosome. We speculate that the interaction of mycolactone with CL6 could change the conformation of these regions and consequently could alter the interaction and/or rate of release. This fits well with the changes in trypsin sensitivity of CL6 and CL7 observed by McKenna et al. [18].

In summary, binding of mycolactone to Sec61α is reminiscent of the ‘foot-in-the-door' mechanism suggested earlier for cotransin and eeyarestatin compounds [34,67]. By stabilising the translocon in an *intermediate* state that is barely permeable to Ca^2+^ ions and/or altering the efficiency with which the Sec61 translocon can cycle through its various structural states, mycolactone promotes an enhanced Ca^2+^ leak from the ER that likely underpins its cytotoxic effects.

## Data Availability

All supporting data in relation to the studies reported here are provided in this manuscript.

## Open Access Statement

Open access for this article was enabled by the participation of University of Surrey in an all-inclusive Read & Publish pilot with Portland Press and the Biochemical Society under a transformative agreement with JISC.

## Abbreviations

AUC: area under the curve
BU: Buruli ulcer
CHX: Cyclohexamide
CL: cytosolic loop
EM: electron microscopy
IONO: iononophore ionomycin
SERCA: sarco/endoplasmic reticulum Ca^2+^-ATPase
TFP: trifluoperazine
TG: thapsigargin
WASP: Wiskott–Aldrich syndrome protein
WT: Wild-type
