# Supplementary material for: Mycolactone enhances the Ca2+ leak from endoplasmic reticulum by trapping Sec61 translocons in a Ca2+ permeable state
Source: Biochem J. 2021 Nov 23;478(22):4005–24. doi: 10.1042/BCJ20210345 (PMC8650850; doi:10.1042/BCJ20210345)
Supplement: Supplementary Figures S1-S4 [file BCJ-478-4005-s1.pdf]

Mycolactone enhances the Ca^{2+} leak from endoplasmic reticulum by trapping Sec61 translocons in a Ca^{2+} permeable state

Pratiti Bhadra, Scott Dos Santos, Igor Gamayun, Tillman Pick, Clarissa Neumann, Joy Ogbechi, Belinda S. Hall, Richard Zimmermann, Volkhard Helms, Rachel E. Simmonds, Adolfo Cavalié

Supplementary Figure

Supplementary Figure S1. Conformations of (a) mycolactone A (Z isomer) and (b) mycolactone B (E isomer).

Supplementary Figure S2. Docking regions in and around human Sec61α for (a) *idle* and *inhibited* states, and (b) for the *open* state.

Supplementary Figure S3. Effects of mycolactone on cytosolic Ca^{2+} transients of RAW 264.7 cells.

Supplementary Figure S4. Effects of the Ca^{2+} leak enhancer trifluoperazine on the Ca^{2+} mobilisation of HCT116 cells.

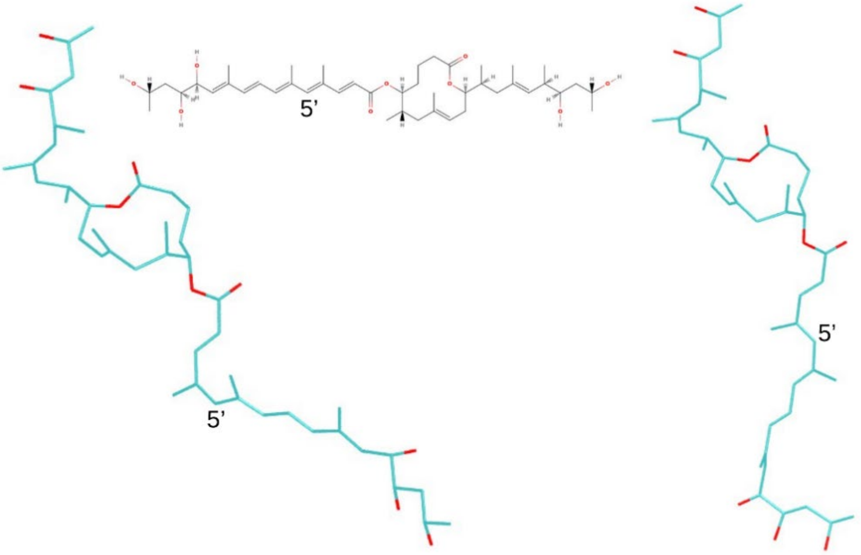

(a) Mycolactone A (Z isomer) (b) Mycolactone B (E isomer)

Supplementary Figure S1. Conformations of (a) mycolactone A (Z isomer) and (b) mycolactone B (E isomer). The images were generated using VMD-1.9.4 and online PubChem Sketcher V2.4.

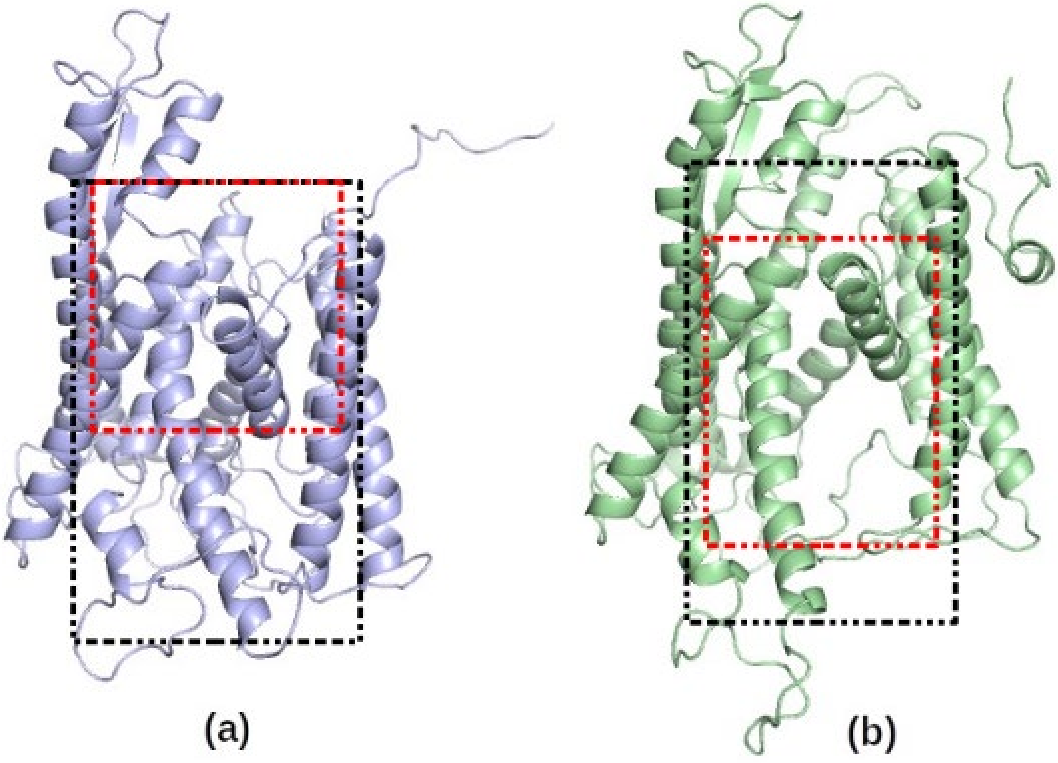

Supplementary Figure S2. Docking regions in and around human Sec61α for (a) *idle* and *inhibited* states, and (b) for the *open* state. The black-dashed boxes represent the grid box (100 Å × 100 Å × 126 Å) used for the first stage of docking and the red-dashed boxes show the grid box used in the second (finer) docking stage (90 Å × 80 Å × 80 Å), (80 Å × 90 Å × 80 Å) and (80 Å × 80 Å × 90 Å) for *idle*, *intermediate,* and *open* state, respectively. The conformations in (a) and (b) are homology models of *idle* and *open* states, respectively.

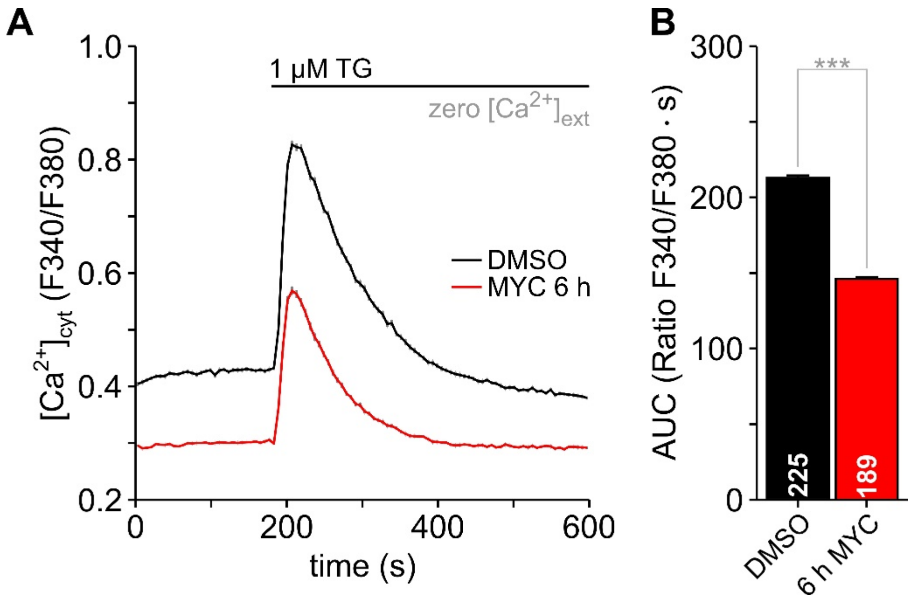

Supplementary Figure S3. Effects of mycolactone on cytosolic Ca^{2+} transients of RAW 264.7 cells. Cytosolic Ca^{2+} was imaged with FURA-2 and changes in $[Ca^{2+}]_{cyt}$ are given as F340/F380 ratios. Cells were treated with 0.05 % DMSO or with 125 ng/ml mycolactone (MYC) for 6 h before FURA-2 loading and Ca^{2+} imaging. Ca^{2+} mobilisation was induced by applying thapsigargin (**A**, 1µM TG). The statistical analysis of the corresponding area under the curve (AUC) for DMSO and MYC treated RAW 264.7 cells is shown in **B**. Data is presented as means ± SEM; ***, $p<0.001$. The number of cells is given within the graph bars in **B**.

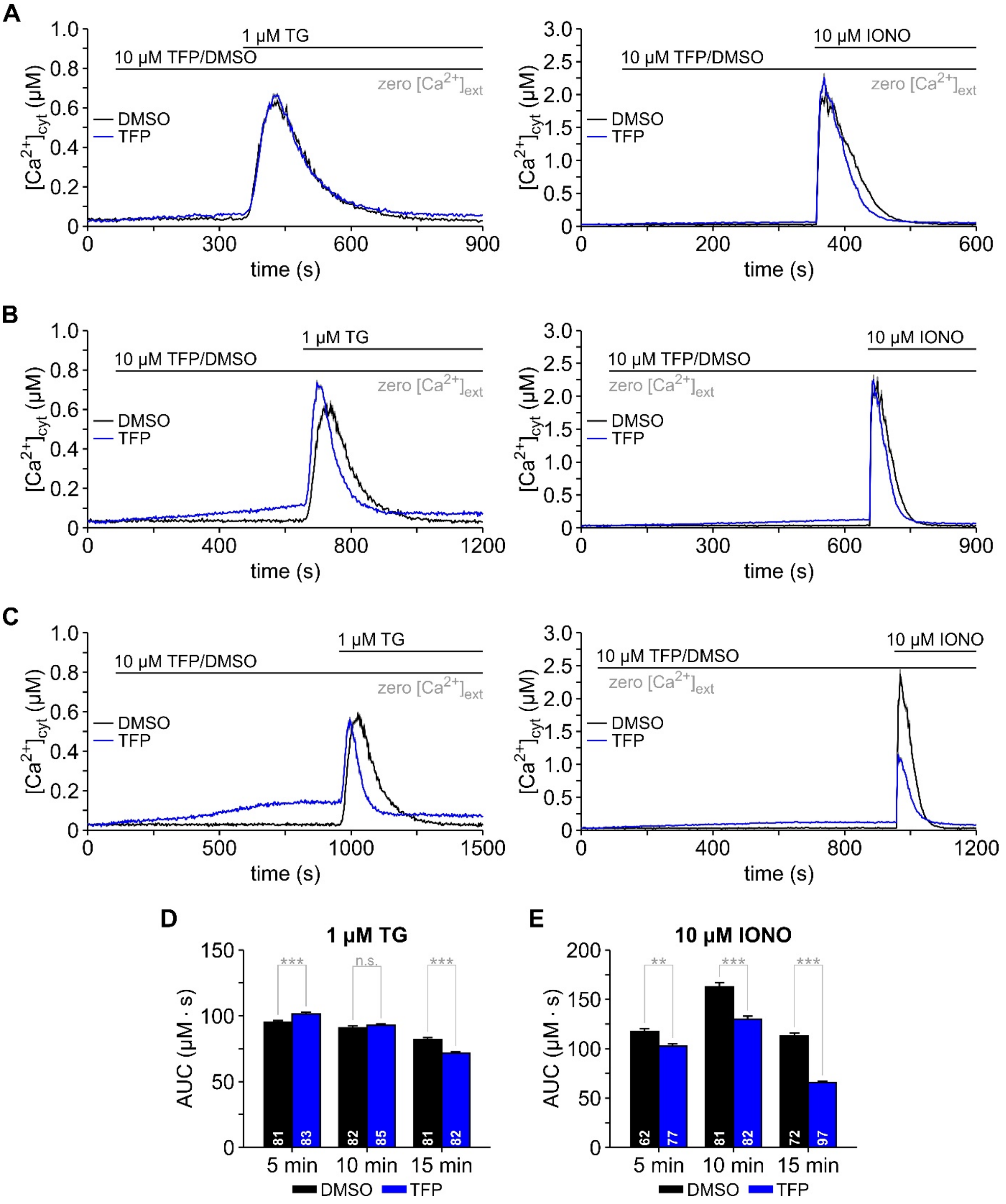

Supplementary Figure S4. Effects of the Ca^{2+} leak enhancer trifluoperazine on the Ca^{2+} mobilisation of HCT116 cells. Trifluoperazine (TFP) was used as a reference substance for comparison with the effects of mycolactone on ER Ca^{2+} leak. $[Ca^{2+}]_{cyt}$ was imaged with FURA-2 in HCT116 cells that were exposed "online" to 10 µM TFP (TFP) or 0.05 % DMSO (DMSO) for 5 min (**A**), 10 min (**B**) and 15 min (**C**). At the end of the mycolactone treatment, Ca^{2+}

mobilisation was induced with thapsigargin (1 µM TG, *left panels*) or with ionomycin (10 µM IONO, *right panels*) to estimate the Ca^{2+} leak from ER and the total Ca^{2+} content in the cells, respectively. TFP effects on TG- and IONO-induced Ca^{2+} transients were quantified as area under the curve (AUC) and are shown in **D** and **E**, respectively. The number of cells is given within the graph bars (**D**, **E**). Data is presented as means ± SEM; n.s., non-significant; **, $p<0.01$; ***, $p<0.001$.
